# Comparing the use of PGMI scoring systems used in the UK and Norway to assess the technical quality of screening mammograms: a pilot study

**DOI:** 10.1186/bcr3296

**Published:** 2012-11-09

**Authors:** M Boyce, R Gullen, D Parashar, K Taylor

**Affiliations:** 1 Cambridge Breast Unit, Cambridge University Hospitals NHS Foundation Trust, Cambridge, UK; 2 Oslo Universitetssykehus, Ullevål, Oslo, Norway; 3 Cambridge Cancer Trials Centre, Cambridge University Hospitals NHS Foundation Trust, Cambridge, UK

## Introduction

The UK and Norway use PGMI scoring to critique mammographic image quality (IQ). PGMI comprises categories with associated criteria for determining mammograms Perfect, Good, Moderate, Inadequate. Implementation of PGMI may be variable, subjective and interpreted locally, making accurate comparison of performance across countries difficult. We compared PGMI use in Cambridge and Oslo, determining differences and possible contributory factors, enabling suggestions for future research and practice.

## Methods

Digital mammograms from 112 consecutively screened women were sourced in each centre. Test sets were enriched with mammograms from each PGMI category and independently scored by four mammographers, each with ≥4 years' experience, using local PGMI. Each image was individually scored P, G, M, or I. Reasons for scoring less than perfect were documented and each mammogram assigned an overall PGMI score. Test sets were exchanged and the process repeated.

## Results

Cambridge uses 17 criteria for scoring mammograms less than perfect. Oslo uses similar criteria, but subcategorised, totalling 39 criteria. There is fair agreement (κ = 0.38) between centres in assigning images as acceptable overall (P, G, M) but poor inter-rater agreement within and between centres in further categorising acceptable mammograms as P, G or M (κ <2). Most common faults in Oslo were skin folds, and inadequate pectoralis muscle in Cambridge. Most faults overall were on oblique views.

## Conclusion

Poor rater agreement and differing faults may be due to the variation in number and interpretation of categories used. Radiographer training may also be an issue. Further research should establish quantitative assessment methods and internationally uniform practice.

